# Pripper: prediction of caspase cleavage sites from whole proteomes

**DOI:** 10.1186/1471-2105-11-320

**Published:** 2010-06-15

**Authors:** Mirva Piippo, Niina Lietzén, Olli S Nevalainen, Jussi Salmi, Tuula A Nyman

**Affiliations:** 1 Department of Information Technology, University of Turku, Turku, Finland; 2 Institute of Biotechnology, University of Helsinki, Helsinki, Finland

## Abstract

**Background:**

Caspases are a family of proteases that have central functions in programmed cell death (apoptosis) and inflammation. Caspases mediate their effects through aspartate-specific cleavage of their target proteins, and at present almost 400 caspase substrates are known. There are several methods developed to predict caspase cleavage sites from individual proteins, but currently none of them can be used to predict caspase cleavage sites from multiple proteins or entire proteomes, or to use several classifiers in combination. The possibility to create a database from predicted caspase cleavage products for the whole genome could significantly aid in identifying novel caspase targets from tandem mass spectrometry based proteomic experiments.

**Results:**

Three different pattern recognition classifiers were developed for predicting caspase cleavage sites from protein sequences. Evaluation of the classifiers with quality measures indicated that all of the three classifiers performed well in predicting caspase cleavage sites, and when combining different classifiers the accuracy increased further. A new tool, Pripper, was developed to utilize the classifiers and predict the caspase cut sites from an arbitrary number of input sequences. A database was constructed with the developed tool, and it was used to identify caspase target proteins from tandem mass spectrometry data from two different proteomic experiments. Both known caspase cleavage products as well as novel cleavage products were identified using the database demonstrating the usefulness of the tool. Pripper is not restricted to predicting only caspase cut sites, but it gives the possibility to scan protein sequences for any given motif(s) and predict cut sites once a suitable cut site prediction model for any other protease has been developed. Pripper is freely available and can be downloaded from http://users.utu.fi/mijopi/Pripper.

**Conclusions:**

We have developed Pripper, a tool for reading an arbitrary number of proteins in FASTA format, predicting their caspase cleavage sites and outputting the cleaved sequences to a new FASTA format sequence file. We show that Pripper is a valuable tool in identifying novel caspase target proteins from modern proteomics experiments.

## Background

Caspases form a protein family of aspartate-specific proteases [1]. Altogether 15 different caspases have been identified in mammals, and they are grouped into two major subfamilies, inflammatory and apoptotic caspases [2]. Caspases mediate their effects through aspartate-specific cleavage of their target proteins, and several hundreds of caspase target proteins have been identified [3]. This caspase-cascade plays a central role in the induction and transduction of apoptotic signals, as well as in the regulation of immunity, cellular proliferation and differentiation [2].

Typically, caspases recognize a four amino acid long motif P_4_P_3_P_2_P_1 _in their target sequences. Most often the last amino acid, P_1_, is Asp (D) but in some rare cases also Glu (E), Gly(G) or Ala (A) [3,4]. Studies of amino acids in the motif have revealed that the motif is only moderately conserved between target proteins of different caspases. Caspases 1, 4, 5 and 13 tend to recognize the motif (W/L)EHD, caspases 2, 3 and 7 DEXD and caspases 6, 8, 9 and 10 the motif (I/L/V)E(H/T)D [4,5]. Although a certain protein might have several caspase cleavage sites, recent studies indicate that it is cut at a single site at a time [6]. The known caspase cut sites do not exhibit any preferences for location in functional domains of proteins [6].

The prediction of caspase cleavage sites from proteins is not a trivial task due to the heterogeneity of target sequences, and several different tools have been developed to predict caspase cleavage sites from individual proteins. PeptideCutter http://www.expasy.org/tools/peptidecutter/, PEPS [7], CasPredictor [8] and GraBCas [9] are based on scoring matrices to score different positions and amino acids at and near the caspase cleavage sites. The specific site in the sequence is either classified as a caspase cut site or as a non-cut site based on the score. In addition, three tools using pattern recognition methods have been developed. They use either support vector machines (CASVM) [4,10] or neural networks [11]. Recently described Cascleave uses the SVM method, but in addition to the primary sequence data of the proteins it also utilizes some structural features to predict caspase cleavage sites [4]. In addition, a two step-model has been suggested that uses first either CASVM or GraBCas and then a scoring method to increase positive predictive value of the classifiers [12]. These prediction tools are valuable in offering a method to predict caspase cut sites for a single protein. However, currently there are no freely downloadable tools that could predict cut sites for several proteins simultaneously, e.g. for entire proteomes in a single run or combine several different methods for prediction. The availability of caspase cut sites for the whole genome could significantly aid in identifying caspase target sequences from tandem mass spectrometry data of biological samples.

We have developed a new tool, Pripper (**Pr**otein sn**ipper**) for predicting caspase cut sites for an arbitrary number of protein sequences. Three different pattern recognition classifiers were trained to predict caspase cut sites. The first is based on the support vector machine [13], the second on random forests [14] and the third on the J48 algorithm [15]. One additional classifier (Vote) which is capable of combining the prediction results from selected classifiers was also constructed in the tool. Pripper is not restricted to predicting only caspase cut sites, but it gives the possibility to scan protein sequences for any given motif(s) and predict cut sites once a suitable cut site prediction model for any other protease has been developed. A newly created classifier can be easily incorporated in the tool with minor effort of programming.

## Implementation

Supervised learning methods were used to form recognizers for caspase cut sites in Pripper. These methods use a training data set of candidate cut sites whose classification is known along with a number of features connected to these candidates. A robust way to teach the classifier is to use the leave-one-out method in which the classifier is taught as many times as there are objects in the training data set. One object is left out from the training set and will be used to test the classifier which is formed on this reduced training set. This way each object is used once for testing and the combined results are used to evaluate the performance of the trained classifier.

### Selection of the training data

The training sequences for known caspase cut sites were acquired from published material [3,6,16]. Other species than human were excluded from the sequence sets due to their smaller number of known cut sites. The sequences were downloaded using the EBI Dbfetch tool http://www.ebi.ac.uk/cgi-bin/dbfetch. It was verified that each of the downloaded sequences contained the published motif at the published cut site.

Altogether, 443 positive cut site samples were gathered from 358 different proteins. Negative training sequences were generated from the same sequences that were used for positive sequences. The 443 negative sites were selected at the positions of Asp (D) that were not detected as caspase cut sites. The used positive and negative sequences are listed in the Supplementary material (Additional file 1: Training set). All the sequences used in the training set were unique.

### Training of the classifiers

Three different pattern recognition classifiers were trained to predict caspase cut sites from proteins. The first classifier was implemented with Support Vector Machines (SVM) [13,17]. SVMs are classifiers that are based on the maximization of the margin between the classes. The data are considered as n-dimensional vectors and the algorithm finds a hyperplane that separates the vectors in different classes with a maximal margin. The SVM method is based on the fact that a kernel function can be used to map vectors of the original feature space to a higher dimensional space in which the data can always be linearly separated. Feature vectors consisted of a fixed number of amino acids on both sides of the cutting site encoded in a numerical form. Each amino acid in the sequence was represented as an array of length 20 representing the 20 different amino acids. Only one element was set to one and the rest to zero and the number one identifies the amino acid in question. For example, the amino acid Ala (A) was encoded by a vector [0, 0, 0, 0, 1, 0, 0, 0, 0, 0, 0, 0, 0, 0, 0, 0, 0, 0, 0, 0] and Val (V) as [1, 0, 0, 0, 0, 0, 0, 0, 0, 0, 0, 0, 0, 0, 0, 0, 0, 0, 0, 0]. A similar encoding has been used in other methods utilizing SVMs [4,10]. The LIBSVM library [18] was used to train the SVM-classifier.

The second trained classifier was the J48-method that is implemented in the Weka classification library [15]. It is a version of the C4.5 algorithm [19] that is a decision-tree-based classifier. The training data are organized as subtrees based on a selected feature that most effectively splits the data set. Information entropy is used to calculate an impurity value and the best split is used to split the tree and the same procedure is iterated until the tree cannot be divided anymore. Typically, also a pruning method is used to remove uninformative nodes of the tree and to improve the prediction accuracy and to avoid over-fitting. The leaves of the trees represent the classification results.

The third method used was Random Forest (RF) that is based on training a collection of trees, called forest, using randomly selected features for splitting the decision tree during the tree growing process [14]. Each sample is classified with each tree in the forest during the classification, and the final output is given as the vote of all the trees. The class that gets the highest vote will be selected as the class output for the sample.

Parameters of the classifiers were optimized using the leave-one-out method (Table 1). The optimization was done for all different sequences varying between 4, 6,..., 16 amino acids to the left and 0, 2,..., 16 amino acids to the right of the cleavage site. The sequence length and parameter combination that produced the best accuracy was selected as the classifier to be used.

**Table 1:** Optimized parameter values for trained classifiers.

Classifier	Parameter	Value
SVM	SVM method	NU-SVC
	Kernel type	RBF
	Kernel function parameter γ	2^-5.5^
	Error parameter ν	0.536
	Stopping criterion ε	0.00001
	Sequence length	10
		6 before cut site
		4 after cut site
Random forest	Maximum depth	unlimited
	Number of features	4
	Number of trees	143
	Sequence length	24
		12 before cut site
		12 after cut site
J48	Confidence factor	0.285
	Minimum number of objects	5
	Number of folds	3
	Binary splits	true
	Reduced error pruning	false
	Subtree raising	true
	Unpruned	false
	Use Laplace	false
	Sequence length	6
		4 before cut site
		2 after cut site

### Classifier evaluation

Each classifier was trained using the leave-one-out method. The number of correct positive classifications (true positives, TP), incorrect positive classifications (false positive, FP), correct negative classifications (true negatives, TN) and incorrect negative classifications (false negatives, FN) were counted from the leave-one-out training. Using these values the following measures for classifier evaluation were calculated.

Accuracy:

Precision:

False discovery rate:

Specificity:

Matthews Correlation Coefficient:

### Pripper

The application for generating the cut sites from the given set of proteins was implemented in Java. The application uses the LIBSVM [18] and Weka version 3.5.7 [15] libraries to load the trained classification models and to use them in the classification of the protein sequences. In addition, each classifier can be used separately to predict the caspase cut sites, or a Vote-classifier can be used. In the Vote-classifier the user can select the desired classifiers and the tool predicts the cut sites with each selected classifier and classifies any site to be a cut site only if the majority of the selected classifiers predict the site as a cut site.

### Protein samples and tandem mass spectrometry analysis

Two different protein samples were used to test the performance of the created caspase cleavage databases in the analysis of tandem mass spectrometry (MS/MS) data. One of the samples was a mitochondrial proteome of human keratinocytes subjected to two-dimensional electrophoresis (2-DE) based proteome analysis [20]. The other sample was a mitochondrial fraction of influenza A virus infected human primary macrophages analyzed using in-solution digestion and iTRAQ-labeling protocol (Applied Biosystems). In both samples, proteins were digested with trypsin and the resulting peptides were analyzed by LC-MS/MS using an Ultimate 3000 nano-LC (Dionex) and a QSTAR Elite hybrid quadrupole TOF-MS (Applied Biosystems/MDS Sciex) with nano-ESI ionization. The LC-MS/MS data were searched with in-house Mascot version 2.1 through ProteinPilot 2.0.1 interface. The Mascot search criteria used were trypsin digestion with one missed cleavage allowed, carbamidomethyl modification of cysteine (2-DE sample) or methylthio modification of cysteine and 4plex iTRAQ labelling of lysine and peptide N-terminus (iTRAQ sample) as fixed modifications, methionine oxidation as variable modification, precursor ion mass tolerance of 50 ppm, fragment ion mass tolerance of 0.2 Da, and peptide charge state of +1, +2 or +3. Identification threshold of p < 0.05 was used with an additional requirement of at least one unique peptide identification for each protein ("bold red").

## Results

### Training and evaluation of the classifiers to predict caspase cleavage sites

Caspases recognize a four amino acid long motif P_4_P_3_P_2_P_1 _in their target sequences; however, the prediction of caspase cleavage sites from proteins is a non-trivial task due to the heterogeneity of the target sequences. Three different classifiers were trained in the present research to predict caspase cleavage sites in protein sequences. Support vector machines have already been widely used and they have turned out to be effective in various biological classification problems including the caspase cleavage site prediction [4,10]. Therefore, it was selected also here as one of the trained classifiers. In addition, two different tree-based classifiers were trained, the Random Forest (RF) and J48. Tree-based methods have been used more rarely in biological classification problems, but RF has shown some promising results in other context [21] and therefore it was selected as one of the trained models. One advantage of the decision-tree based models is that they output the decision tree and show the exact decision rules to the user. Since RF makes its decision on the basis of several trees, it can not give a single and easily readable decision tree as an output. Therefore, also a pure decision tree model, J48, that is capable of producing a single decision tree as an output was selected as one of the classifiers.

Training of the classifiers produced the best sequence length to be used in the prediction for each classifier. The best protein sequence length differed between the classifiers from 4 to 12 amino acids before and 2 to 12 amino acids after the cut sites. The best sequence length for the SVM classifier was 6 amino acids before the cut site and 4 after it, and therefore the trained classifier was named SVM-6-4. Respectively, the two other classifiers are called RF-12-12 and J48-4-2. It is noteworthy that the different classifiers have different optimized lengths of protein sequences indicating that the amino acids surrounding the cut sites are very heterogeneous. Based on these data and previously published results, it is clear that the reliable prediction of caspase cleavage sites requires information of several amino acids surrounding the motif. The optimized lengths were quite similar to the best CASVM classifier that takes into account 14 amino acids before and 10 after the cut site (CASVM P14-P10') [10]. CASVM has also been optimized for shorter lengths containing only the cut site motif (P4-P1) or the cut site motif and the two following amino acids (P4-P2').

**Figure 1:**
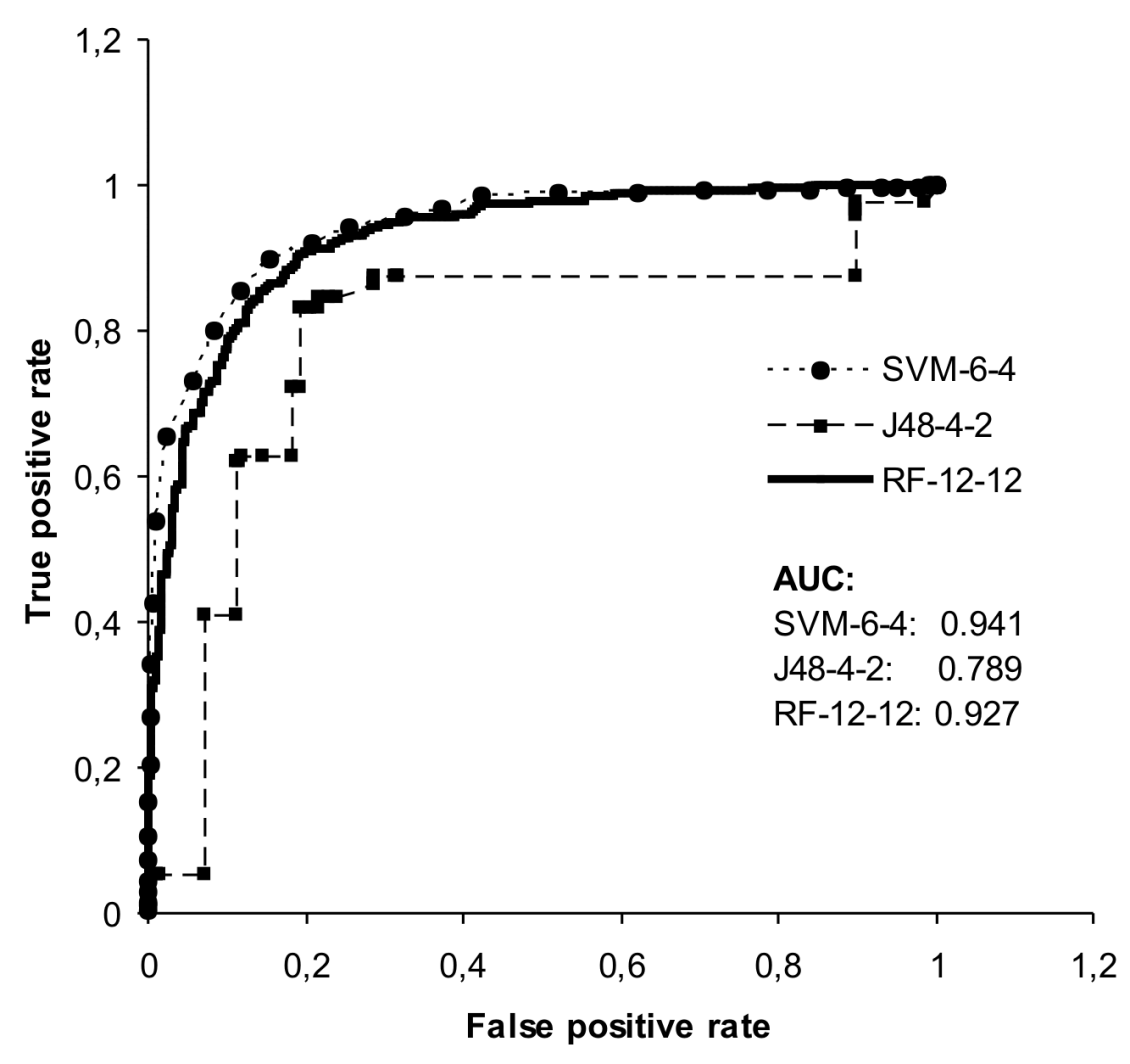
**The ROC curves of the trained classifiers**. Area under ROC curve (AUC) is highest for SVM-6-4 and lowest for J48-4-2, and thus SVM-6-4 and RF-12-12 show better performance than the J48-4-2 classifier.

An ROC curve was constructed for the trained classifiers using different threshold values. The ROC curve visualizes the performance of a classifier by plotting the false positive rate versus the true positive rate while varying the threshold for classification result [22]. The best threshold value for a classifier is achieved when the false positive rate is as small as possible and at the same time the true positive rate is as high as possible. Therefore the best values are located at the top left corner of the ROC curve. The ROC curves of the trained classifiers show that all of the trained classifiers were good (Figure 1). The curves of SVM-6-4 and RF-12-12 were very similar, the curve of SVM-6-4 being slightly above the RF-12-12 curve. The J48-4-2 curve stayed below the curves of the other classifiers indicating the weakest performance for this classifier as compared to the other two classifiers. The best classifiers were SVM-6-4 and RF-12-12 as evaluated by the area under ROC curve (AUC). The AUC for RF12-12 and SVM-6-4 were 0.927 and 0.941, respectively.

Although the J48 classifier was not the most efficient, the decision tree can reveal important insight for caspase cleavage sites (Additional File 2 - J48-4-2 Decision Tree). The J48-4-2 classifier was trained with the sequence length of 4 amino acids before the cut site and 2 amino acids after the cut site (X_1_X_2_X_3_X_4_|X_5_X_6_). The decision tree shows that the classifier uses information from 5 amino acids. The most important features for the prediction are the amino acid that follows the cut site (X_5_), and the first amino acid in the beginning of the cut site motif (X_1_). The last residue of the cut site motif, X_4_, is not part of the tree, but this can be explained by the fact that all negative samples were designed to contain Asp (D) in that position leaving this residue uninformative.

### Comparison of the trained classifiers with existing caspase cleavage prediction models

The true positive and negative rates and false positive and negative rates were calculated from the leave-one-out training results to acquire the quality measures, namely accuracy, precision, false discovery rate, specificity and Matthews correlation coefficient. The used sequences were also classified with the publicly available tools for caspase cut site prediction PeptideCutter, CASVM and GraBCas (Table 2). Since we were not able to get any result output files from the recently published Cascleave, it could not be taken for comparison. The results showed that the two classifiers trained in this study, SVM-6-4 and RF-12-12, and the earlier published classifier CASVM P14-P10' [10] were the most accurate classifiers when measured by the accuracy of classified test sequences (Table 2). Also the Matthews correlation coefficient showed that the actual and predicted results correlated best in these three classifiers. When the SVM-6-4 and RF-12-12 classifiers were combined in the Vote-classifier, an increase in the quality of classification was clearly seen as an increase in all other quality measures except in false discovery rate which decreased substantially. We also confirmed the test results by performing additional tests for the J48-4-2, RF-12-12 and SVM-6-4 classifiers by first using 2/3 of the data set with the leave-one-out method as described above, and then testing with 1/3 of the data set. The results of this test agreed with the results in Table 2, the test set accuracies being 77,9%, 83,1% and 84,1%, respectively.

**Table 2:** Quality measures of trained classifiers and comparison with publicly available caspase cut site prediction models.

Classifier	ACC	PRC	FDR	SPC	MCC
SVM-6-4	87,4%	85,3%	14,7%	84,4%	74,8%
RF-12-12	85,7%	82,5%	17,5%	82,5%	71,7%
J48-4-2	81,6%	79,7%	20,3%	78,3%	63,3%
Vote	96,8%	100%	0,0%	100%	93,7%
PeptideCutter	50,8%	63,0%	37,0%	97,7%	4,6%
GraBCas	67,7%	67,6%	32,4%	67,5%	35,4%
CASVM P4-P1	62,3%	83,0%	17%	93,7%	31,6%
CASVM P4-P2'	72,7%	73,1%	26,9%	73,6%	45,4%
CASVMP14-P10'	83,1%	81,6%	18,4%	80,1%	66,2%

Peptide cutter, GraBCas and CASVM were all tested with the complete training data. The values obtained from SVM-6-4, J48-4-2 and RF-12-12 represent the values obtained from the test sequences of the training with the leave-one-out method. The default cut off value 1.2 was used in GraBCas. Classifiers RF-12-12 and SVM-6-4 were used in the Vote classifier. Abbreviations: ACC, accuracy; PRC, precision; FDR, false discovery rate; SPC, specificity; MCC, Matthews correlation coefficient.

**Figure 2:**
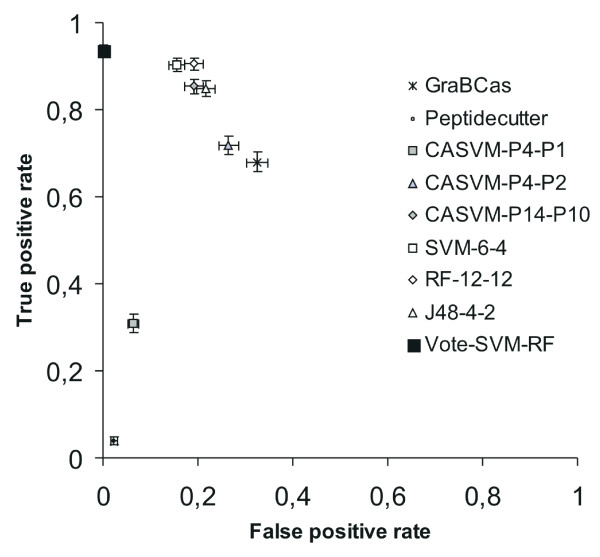
**ROC curve comparisons of caspase cleavage site classifiers**. The data points represent the average True positive and False positive rates from the 10 000 bootstrap values. The lines surrounding the data points represent the standard deviation calculated from the bootstrap data. The ROC curve shows that the Vote classifier that combined the RF-12-12 and SVM-6-4 classifiers appears to be the best among the compared classifiers, since its True positive rate is very close to one and False positive rate is zero.

The trained classifiers and the publicly available classifiers were compared using an ROC curve (Figure 2). 10 000 bootstrap datasets were created by sampling the original test set with replacement to form 10 000 new test sets of similar size as the original test set. Each classifier classified all of the 10 000 bootstrap data sets and from each set the true and false positive rates were calculated, averaged and plotted to an ROC curve. In this case the threshold values are not varied in the classifiers, but each classifier classified the sequences by their default, optimized threshold value. Therefore each classifier is represented as a single point, and the multiple values from the bootstrap sets provided the standard deviation estimates. A comparison of different classifiers shows that the best classifiers are the SVM-6-4, RF-12-12 and Vote since they are located to the most upper left corner in the graph (Figure 2).

### Developing Pripper, an application for predicting caspase cut sites from arbitrary number of protein sequences

A new tool, Pripper, was developed to read an input file of protein sequences, to scan each protein sequence and predict cleavage sites for each protein and to write the predicted cleavage products to a new data file. Pripper reads input data and outputs a data file of protein sequences in FASTA format. The user can choose the motifs that the sequences are searched for, and classification is performed only to those sequence regions. If the user does not give any motifs, Pripper scans the whole length of the protein sequence residue by residue and performs the classification.

The user can also choose whether all possible cleavage product combinations (cut at single or multiple sites) are written to the output file, or whether each of the sequences is cut from a single site at a time or from all of the predicted sites simultaneously. User can also choose whether the full sequence is written to the output file or not. The tool allows also a list of known cut sites to be used instead of the prediction. If a protein has user-given cleavage sites, new cleavage sites are not predicted from the protein but the given sites are used instead, and the cleavage products are written to the output file based only on those given cut sites. Pripper is freely available at http://users.utu.fi/mijopi/Pripper.

### Estimation of caspase cut sites from human protein sequences

We estimated the number of caspase cleavage sites in human proteome using Pripper with the trained caspase cleavage site classifiers. All human protein sequences were downloaded from the UniProt database UniProtKB that contains both carefully annotated SwissProt entries as well as automatically annotated TrEMBL entries. Pripper was used to predict cut sites for this dataset, and the predicted cut sites in individual proteins were counted (Table 3). Asp (D) amino acid was selected as the motif for the prediction.

**Table 3:** Predicted caspase cut sites of the human protein sequences downloaded from UniProtKB database.

	SVM-6-4	RF-12-12	J48-4-2	Vote
Predicted number of cut proteins	70 343	73104	79 926	66 513
% of all proteins (96 123)	73,2	76,1	83,1	69,2
Average number of cut sites per sequence	4,3	4,8	5,9	3,7
Predicted cut sites total	301 678	348 600	468 865	246 692
% of all possible cut sites (1 749 441)	17,2	19,9	26,8	14,1

The cut sites were predicted with Pripper using Vote classification (RF-12-12 and SVM-6-4). The cut sites were predicted only from positions that had Asp (D) in the cut site; the given number of all possible cut sites is the number of Asp (D) residues in the protein sequences.

SVM-6-4 predicted that 73,2% of all the proteins contained a cut site. RF-12-12 predicted a roughly similar amount of 76,1%, but J48-4-2 predicted that more than 80% of the proteins contained a cut site. The Vote-classifier was selected to perform the classification with both SVM-6-4 and RF-12-12, and it predicts a cut site only if both classifiers predicted the site to be cut. This classifier predicted approximately 69% of the proteins to have at least one caspase cleavage site. Based on this analysis, the human proteome contains approximately 66 500 caspase targets. The cleavage products from human protein sequences that were predicted by the Vote-classifier are available in Additional file 3.

Currently, only a few hundred caspase target proteins are known [3,10], but it has been suggested that the cells could contain thousands of caspase targets [8]. Since the trained classifiers are based on the primary sequence data of proteins, it is possible that the cells contain putative caspase target sites, but they reside in the protein regions that are not accessible to caspases when the protein has folded to its three-dimensional structure. The caspase cut sites are not linked to any functional protein domains [6] However, it has been discovered that many of the caspase cleavage sites occur in loops and in α-helical regions [6], as well as in PEST-regions [8]. PEST-regions are rich in Ser (S), Thr (T), Pro (P), Glu (E), and Asp (D) and actually form non-structural regions that are often found in unstable proteins that are susceptible to proteolysis [23]. Therefore, the prediction efficiency of the classifiers might be improved by taking into account the secondary or higher-order structure of proteins.

### Analysis of tandem mass spectrometry data with a sequence database containing caspase cleavage products

**Figure 3:**
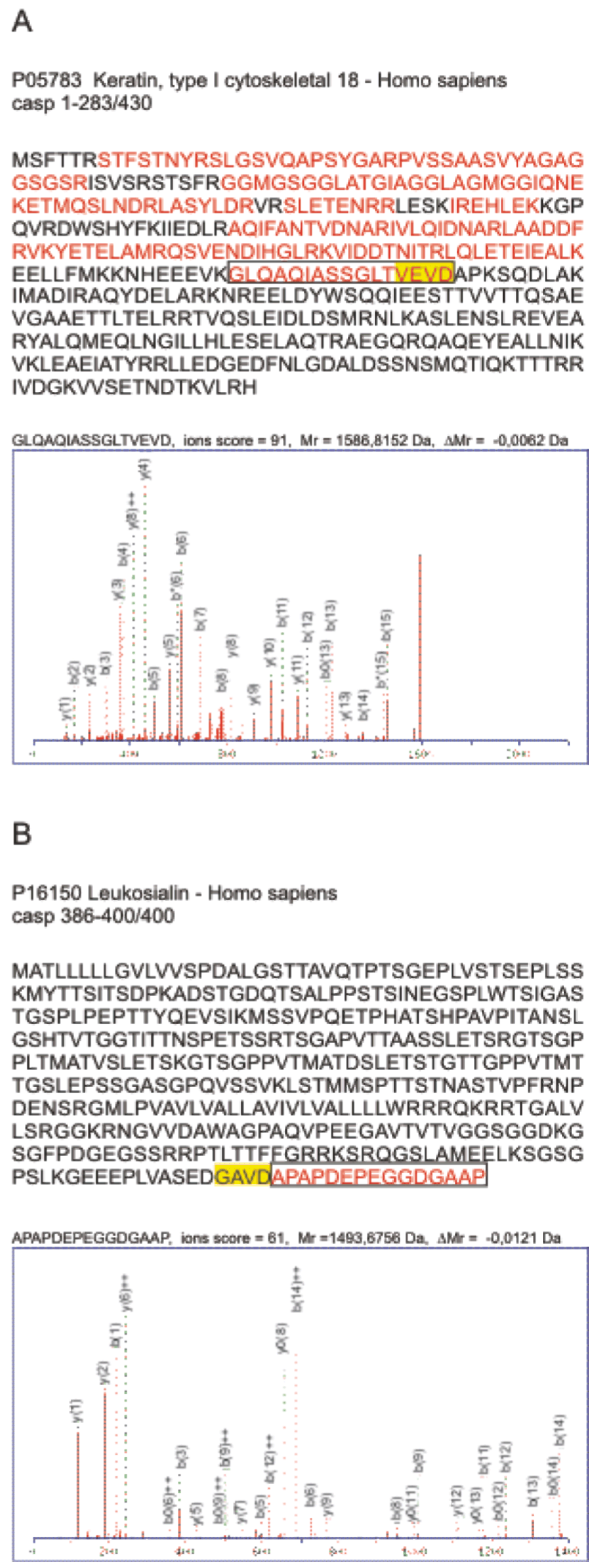
**Caspase cleavage products identified based on MS/MS-data**. Protein identifications were done with Mascot against a database of predicted caspase cleavage products created with Pripper (Vote-classifier: SVM-6-4 and RF-12-12). Complete protein sequences are shown with all the identified peptides (red), caspase cleavage motifs (yellow) and peptides identified at the cleavage site (black box). Peptide spectrum matches for the cleavage site peptides are also shown. A) Cytokeratin 18 identified from the 2-DE sample B) Potential new caspase target leukosialin identified from the iTRAQ-sample.

Pripper was used to create a sequence database containing both intact protein sequences and predicted caspase cleavage products from SwissProt human protein sequences and ssRNA negative-strand virus protein sequences (both from the release 57.12, 12/09). The database was created using Vote-classifier with SVM-6-4 and RF-12-12, cutting each sequence from only one place at a time and using Asp (D) as the cut site motif. The database was used to identify caspase cleavage products from MS/MS data obtained from two different proteomic experiments. Caspase cleavage product identification was considered reliable only if at least the other one of the peptides at the cleavage site was reliably identified. In the 2-DE based experiment each LC-MS/MS run contained peptides from only one or few proteins located in the same spot in the 2 D gel. Here, one of the protein spots was identified as the N-terminal fragment of caspase-cleaved cytokeratin 18 (Figure 3A) with the already known cut site motif VEVD. In the second experiment the analyzed sample was a complex mixture resulting in the identification of more than 700 proteins. Here we found several potential caspase cleavage products and identified e.g. the known cut sites of beta-actin (P60709, cut site motif ELPD) and myosin (P35579, cut site motif DTLD). Additionally, a potential new caspase cleavage product, leukosialin (P16150), was identified with a cut site motif GAVD (Figure 3B). The ability to use a specific database instead of e.g. searching the data against semi-tryptic cleavages is a great advantage especially with large datasets. The number of semi-tryptic, potential caspase cleavage site peptides is much smaller when a caspase cleavage product database is used, decreasing the amount of manual validation of the results and also making the identification results more easily interpretable.

## Conclusions

We have developed a new tool, Pripper, for reading an arbitrary number of proteins in FASTA format, predicting their caspase cleavage sites and outputting the cleaved sequences to a new FASTA format sequence file. The sequence file generated can be used e.g. as a database for searching tandem mass spectrometry data allowing the identification of caspase cleavage products. Three different pattern recognition classifiers, SVM-6-4, RF-12-12 and J48-4-2 were trained to predict caspase cleavage sites. The evaluation of the classifiers with ROC curve (Figure 1) and quality measures (Table 2) indicated that all of the three classifiers had a good performance for predicting caspase cleavage sites. The comparison of our method to existing caspase cleavage classifiers showed that the best performing classifiers were SVM-6-4, RF-12-12 and Vote developed in this study and the previously published CASVM classifier with the longest prediction sequence (P14-P10') [10].

The developed tool was used to construct a database of caspase cleavage products. Each possible Asp (D) amino acid and its surrounding amino acids were evaluated with SVM-6-4 and RF-12-12 classifiers and a site was predicted as cleaved only if both of the methods predicted the site to be a caspase cleavage site (Vote-classifier in the tool). The created database contains the predicted caspase cleavage products, and it was used to identify caspase cleavage products from tandem mass spectrometry data from real biological samples. Here we have shown that Pripper is a valuable tool in identifying novel caspase target proteins from mass spectrometry based proteomics experiments.

Being a standalone application, Pripper does not rely on web-connections or depend on server availability, and thus the local machine determines the maximum input file size and processing capacity. In addition, it is capable of processing only user given motif patterns from the input sequences and producing the desired cleavage products. It enhances the prediction results by combining different classifiers. In addition, the implemented application is not restricted to predicting caspase cut sites, but it also provides a framework for scanning protein sequences for given motif for any protease cut sites once a suitable cut site prediction model for a protease is developed.

## Availability and Requirements

**Project name**: Protein Snipper (Pripper)

**Project home page**: http://users.utu.fi/mijopi/Pripper/

**Operating system(s)**: Windows

**Programming language**: Java

**Other requirements**: Java version 1.6.0 or higher, libsvm, Weka, BioJava

**Any restrictions to use by non-academics**: none

## Abbreviations

RF: Random Forest; SVM: Support Vector Machine

## Authors' contributions

MP trained the classifiers and implemented Pripper. NL performed the mass spectrometry analysis and Mascot searches. TN formulated the concept and design of the research idea. MP, NL and TN wrote the paper. ON and JS helped in selection and learning of pattern recognition classifiers and reviewed the manuscript critically. All authors read and approved the final manuscript.

## Supplementary Material

Additional file 1 **Training set**. The set of unique caspase cleavage sites used in the training of the models. Click here for file

Additional file 2 **J48-4-2 Decision Tree**. The training sequences were 6 amino acids long. The attributes 1-4 (att1-att4) belong to the four amino acid long caspase cut site motif. The rest of the attributes (att5 and att6) represent the amino acids after the cut site. The rectangular boxes indicate the classification results: 1 is positive (cut site) and -1 is negative (not a cut site) classification. Click here for file

Additional file 3 **Caspase cleavage products from human proteome**. Human sequences that were downloaded from the UniProtKB database were cleaved with the Vote classifier combining the SVM-6-4 and RF-12-12 classifiers. Click here for file
