# Supplementary material for: Pripper: prediction of caspase cleavage sites from whole proteomes
Source: BMC Bioinformatics. 2010 Jun 15;11:320. doi: 10.1186/1471-2105-11-320 (PMC2893604; doi:10.1186/1471-2105-11-320)

Additional file 2 J48-4-2 Decision Tree.
The training sequence used was 6 amino acids long. The attributes 1-4 (att1-att4) belong to the four amino acid long caspase cut site motif. The rest of the attributes (att5 and att6) represent the amino acids after the cut site. The rectangular boxes indicate the classification results: 1 is positive (cut site) and -1 is negative (not a cut site) classification.

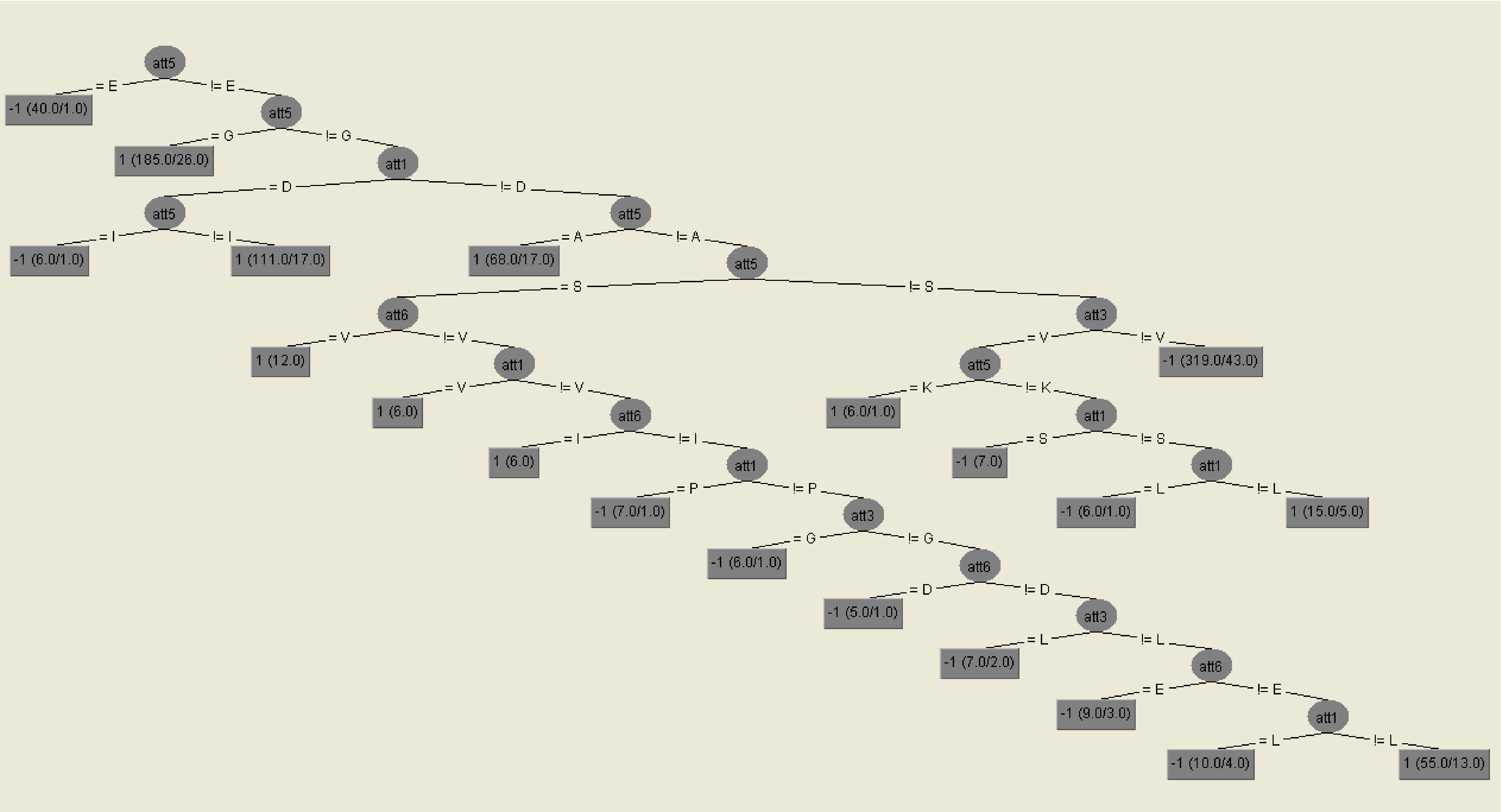

Supplement: Additional file 2 — J48-4-2 Decision Tree. The training sequences were 6 amino acids long. The attributes 1-4 (att1-att4) belong to the four amino acid long caspase cut site motif. The rest of the attributes (att5 and att6) represent the amino acids after the cut site. The rectangular boxes indicate the classification results: 1 is positive (cut site) and -1 is negative (not a cut site) classification. [file 1471-2105-11-320-S2.PDF]
