# Supplementary material for: Pripper: prediction of caspase cleavage sites from whole proteomes
Source: BMC Bioinformatics. 2010 Jun 15;11:320. doi: 10.1186/1471-2105-11-320 (PMC2893604; doi:10.1186/1471-2105-11-320)
Supplement: Additional file 3 — Caspase cleavage products from human proteome. Human sequences that were downloaded from the UniProtKB database were cleaved with the Vote classifier combining the SVM-6-4 and RF-12-12 classifiers. [file 1471-2105-11-320-S3.DOC]

### Additional file 3 – Caspase cleavage products from human proteome can be downloaded from http://users.utu.fi/mijopi/Pripper/uniprot-organism__HomoSapiens.fasta.Vote-SVM-RF.zip
